# HER2-IHC-40x: A high-resolution histopathology dataset for HER2 IHC scoring in breast cancer

**DOI:** 10.1016/j.dib.2025.111922

**Published:** 2025-07-23

**Authors:** Md Serajun Nabi, Mohammad Faizal Ahmad Fauzi, Zaka Ur Rehman, Hezerul Bin Abdul Karim, Phaik-Leng Cheah, Seow-Fan Chiew, Lai-Meng Looi

**Affiliations:** a Faculty of Artificial Intelligence and Engineering, Multimedia University, Persiaran Multimedia, 63100 Cyberjava, Malaysia; b Centre for Image and Vision Computing, COE for Artificial Intelligence, Multimedia University, 63100 Cyberjaya, Malaysia; c Department of Pathology, University of Malaya Medical Centre, 59100 Kuala Lumpur, Malaysia

**Keywords:** HER2 IHC dataset, Breast cancer, Color histogram, Whole slide imaging (WSI), Medical image dataset, Digital pathology

## Abstract

The HER2-IHC-40x and HER2-IHC-40x-WSI datasets are high-resolution whole slide image (WSI) and patch-extracted region collection for HER2 immunohistochemistry (IHC) scoring in breast cancer pathology. 107 WSIs are scanned at 40 × magnification with Regions of Interest (ROIs) annotated by expert pathologists. Patches of 1024 × 1024 pixels are extracted from the ROIs and classified into four HER2 scores (0, 1+, 2+, 3+), yielding structured data for computational pathology analysis. There were two strategies of splitting: WSI-based split, where data was first split before extracting the patches and named as HER2-IHC-40x for this dataset, the other one is patch-based split, where patches were extracted first and then split, named as HER2-IHC-40x-WSI of this dataset. The filtering method for color histograms was applied to remove the non-tumour regions and artifacts, generating high-quality data. The dataset is applicable to deep learning applications, including HER2 classification and explainable AI. It is freely available on Zenodo, with preprocessing scripts provided via GitHub, enabling reproducibility in digital pathology research.

Specifications Table

**Table:** 

Subject	Medical Imaging / Histopathology
Specific subject area	Breast cancer histopathology - HER2 immunohistochemistry (IHC) scoring - Deep learning-based HER2 classification - Whole Slide Imaging (WSI) analysis - High-resolution pathology image processing
Data format	Image (.jpg)
Type of data	Raw, Filtered, Analysed
Data collection	The HER2-IHC-40x dataset was collected from the University of Malaya Medical Centre (UMMC). 107 Whole Slide Images (WSI) of 40 × magnification was scanned on a high-resolution digital pathology scanner. Tumor regions were manually annotated by experienced pathologists to extract Regions of Interest (ROI). Patches of 1024 × 1024 pixels were extracted from the ROIs. Color histogram filtering was applied during preprocessing, removing non-tumour patches. The data was split in two ways: (1) WSI-based 80-20 split prior to patch extraction, and (2) Patch-based 80-20 split post-patch extraction.
Data source location	The data were collected from the following location: Institution: University of Malaya Medical Centre (UMMC) City/Region: Kuala Lumpur Country: Malaysia
Data accessibility	Repository name: HER2-IHC-40x: High-Resolution Histopathology Image Dataset for HER2 Scoring in Breast Cancer Data identification number: 10.5281/zenodo.15179607 Direct URL to data: https://zenodo.org/records/15179608
Related research article	Title: Enhancing HER2 IHC Scoring Using HRNet and SwinT with Cross-Dataset Generalization Authors: Md Serajun Nabi, Mohammad Faizal Ahmad Fauzi, Hezerul Bin Abdul Karim, et al. Journal/Preprint Server: (To be confirmed) URL: (To be provided)

## 1 Value of the Data

• Comprehensive HER2 IHC Dataset for Deep Learning:
  The HER2-IHC-40x dataset provides high-resolution annotated histopathology image patches for HER2 immunohistochemistry (IHC) scoring extracted from whole slide images (WSI) of human breast cancer biopsies. The dataset is beneficial for training deep learning and machine learning models for automated HER2 classification, which can support the diagnosis and treatment planning for breast cancer.
• Facilitates Benchmarking and Model Comparison:
  The dataset allows researchers to evaluate and compare state-of-the-art deep learning models for HER2 scoring in histopathology. The dataset can be partitioned into two halves (WSI-based and Patch-based), promoting cross-domain generalization and dataset bias.
• Supports Explainable AI and Decision-Making in Pathology:
  The dataset offers expert-annotated HER2 classes (0, 1+, 2+, 3+) and is therefore suitable for explainable AI research. By employing Grad-CAM, SHAP, or attention-based models, researchers can investigate how AI models perceive HER2 staining patterns and enhance trust in clinical AI systems.
• Facilitates Reproducibility and Generalization Studies:
  The dataset can be used to study domain adaptation, color normalization techniques, and transfer learning between different histopathology datasets, resulting in digital pathology AI application standardization.

## 2 Background

Breast cancer is one of the leading causes of cancer-related deaths globally. The human epidermal growth factor receptor 2 (HER2) gene play a central role in the breast cancer development process, its amplification or overexpression corresponding to more aggressive forms of breast cancer [1]. HER2 immunohistochemistry (IHC) is a standard test applied to the evaluation of levels of HER2 expression in breast cancer tissues, to determine treatment [2]. While it is of clinical importance, manual assessment of HER2 expression by pathologists is subjective and labor-intensive, which leads to variability [1].

Over the last few years, digital pathology and deep learning have shown promise in automating HER2 expression analysis, and this has improved diagnostic accuracy and efficiency. The whole Slide Imaging (WSI) using high-resolution digital scanning of tissue samples enables computational tools to assist pathologists in diagnosing and classifying tissue samples [3]. However, there is restricted availability of high-quality, annotated datasets for training AI models for HER2 classification.

To bridge this gap, the HER2-IHC-40x dataset was created from high-resolution WSI scans and expert-annotated Regions of Interest (ROIs) from breast cancer tissues. The dataset includes 1024 × 1024-pixel patches cropped from the ROIs, annotated into four HER2 classes: 0, 1+, 2+, and 3+. This dataset is particularly suited for use in deep learning and AI-based HER2 scoring models, presenting a valuable asset for the development of automated diagnosis tools that will be able to assist pathologists in HER2 evaluation and drive improved clinical results.

## 3 Data Description

HER2-IHC-40x, where ‘HER2’ stands for Human Epidermal Growth Factor Receptor 2, ‘IHC’ stands for immunohistochemistry, and ‘40x’ is for dataset magnification. The HER2-IHC-40x dataset is a high-resolution histopathology image dataset for HER2 IHC scoring in breast cancer. The dataset contains only image files and does not include structured metadata (CSV/TXT). The images are systematically categorized into Whole Slide Images (WSI) and extracted patches, ensuring an organized structure for deep learning applications.

### 3.1 Dataset organization

The dataset follows a hierarchical structure to maintain clarity in data processing and experimental reproducibility.

The HER2-IHC-40x dataset is organized hierarchically into three main levels:

• Whole Slide Images (WSI) – Original high-resolution slides.
• Regions of Interest (ROI) – Tumor regions extracted from WSIs.
• Patches – Small image sections (1024 × 1024 pixels) obtained from ROI for AI models.

### 3.2 Description of dataset components

**T**he structure of the files and folders in the dataset is summarized in Table 1. However, only the patch-level data was made publicly available for the training and evaluation of AI models.

**Table 1:** Dataset components and their descriptions.

Folder Name	Content	Description
WSI	.svs (Whole Slide Images)	Contains 107 whole slide images (WSI) scanned at 40 × magnification. These represent the original raw pathology slides before any processing.
ROI	.png (Regions of Interest)	Expert pathologists annotated tumor regions in WSIs. These Regions of Interest (ROI) are extracted before patch generation.
Patches	.png (Image Patches)	Contains 1024 × 1024-pixel image patches extracted from tumor regions of ROIs. Patches are categorized into four HER2 classes (0, 1+, 2+, 3+).
Train	.png (Training Images)	Contains 80% of patches for training AI models.
Test	.png (Testing Images)	Contains 20% of patches reserved for independent evaluation.

### 3.3 HER2 classification and labeling

Each image in the dataset is categorized into one of the four HER2 classes based on immunohistochemical staining intensity [4].

Each WSI, ROI, and filtered patch from Table 1 is assigned to its corresponding HER2 score as shown in Table 2, ensuring clear class labeling.

**Table 2:** HER2 classification and labelling based on staining intensity.

HER2 Class	Description
0 (Negative expression)	No observable HER2 staining.
1+ (Low expression)	Weak, incomplete membrane staining in ≤10% of tumor cells.
2+ (Equivocal expression)	Moderate, circumferential membrane staining in >10% of tumor cells.
3+ (Positive expression)	Strong circumferential membrane staining in >10% of tumor cells.

### 3.4 Preprocessing and quality control

The HER2-IHC-40x data was systematically preprocessed for data relevance, quality, and consistency to HER2 IHC classification, Table 3. These were applied in a uniform manner to all images before any model-specific modifications in order for the dataset to remain cross-purpose for a range of research objectives.

**Table 3:** Overview of preprocessing steps applied to HER2 IHC dataset.

Preprocessing Step	Description
ROI Selection	Expert pathologists manually delineated tumor regions inside Whole Slide Images (WSI), with an eye towards extracted patches containing biologically relevant areas.
Color Histogram Filtering	Non-tumor and background regions were discarded using hue-based histogram correlation (threshold ≥ 0.7) such that the extracted patches will predominantly consist of tumor regions.
Image Normalization	Normalized pixel intensity histograms across all images to reduce staining and illumination variations among different WSIs.
Patch Extraction	1024 × 1024-pixel image patches were extracted systematically from tumor-bearing ROIs in a manner that each patch relates to relevant histopathological structures.

### 3.5 Dataset splitting strategies

The dataset is divided into training (80%) and testing (20%) subsets at the patch level:Table 4

**Table 4:** Dataset splitting strategies and subsets.

Splitting Strategy	Training Set	Testing Set	Total Count
WSI-based Split	86 WSI (8093 Patches)	21 WSI (1847 Patches)	9,940 patches
Patch-based Split	8,897 patches	2,200 patches	10,997 patches

In Table 3, Whole Slide Images (WSIs) were divided before patch extraction; thus, the number of patches varies per WSI, and the total number may not be equal to the Patch-based Split. On the other hand, initially, all the patches were extracted and then split at the patch level in an 80-20 manner, with a consistent total of 10,997 patches. Therefore, the number of patches differs between the two methods due to variation in tissue area per WSI.

### 3.6 WSI’s and patches-based data

As we have formatted the dataset into two categories, the WSI’s based dataset distribution shown in Table 5, call as HER2-IHC-40x, and the patches-based dataset distribution shown in Table 6, call as HER2-IHC-40x-WSI.

**Table 5:** WSI-based data distribution.

Total dataset							
Class	WSI	ROI	Patches				
0	23	429	3789				
1+	26	131	2153				
2+	27	483	634				
3+	31	156	3364				
Total	107	1199	9940				

		
Train Data				Test Data			
Class	WSI	ROI	Patches	Class	WSI	ROI	Patches

0	18	343	3131	0	5	86	658
1+	21	105	1837	1+	5	26	316
2+	22	386	523	2+	5	97	111
3+	25	125	2602	3+	6	31	762
Total	86	959	8093	Total	21	240	1847

**Table 6:** Patch-based data distribution.

Total dataset			
Class	WSI	ROI	Patches
0	23	429	3789
1+	26	131	2689
2+	27	483	1131
3+	31	156	3388
Total	107	1199	10997

		
Train Data		Test Data	

0	3031	0	758
1+	2151	1+	538
2+	905	2+	226
3+	2710	3+	678
Total	8797	Total	2200

## 4 Experimental Design, Materials and Methods

The HER2-IHC-40x dataset was constructed from whole slide images (WSI) of HER2-stained breast cancer tissue samples that were scanned and made ready for computational processing. The dataset was constructed in pursuit of enabling automated HER2 immunohistochemistry (IHC) scoring and deep learning-driven classification. The slides used were sourced from the University of Malaya Medical Center (UMMC) and went through a coordinated pipeline of digitization, region extraction, and dataset organization.

### 4.1 Whole slide imaging and digitization

**Fig. 1:**
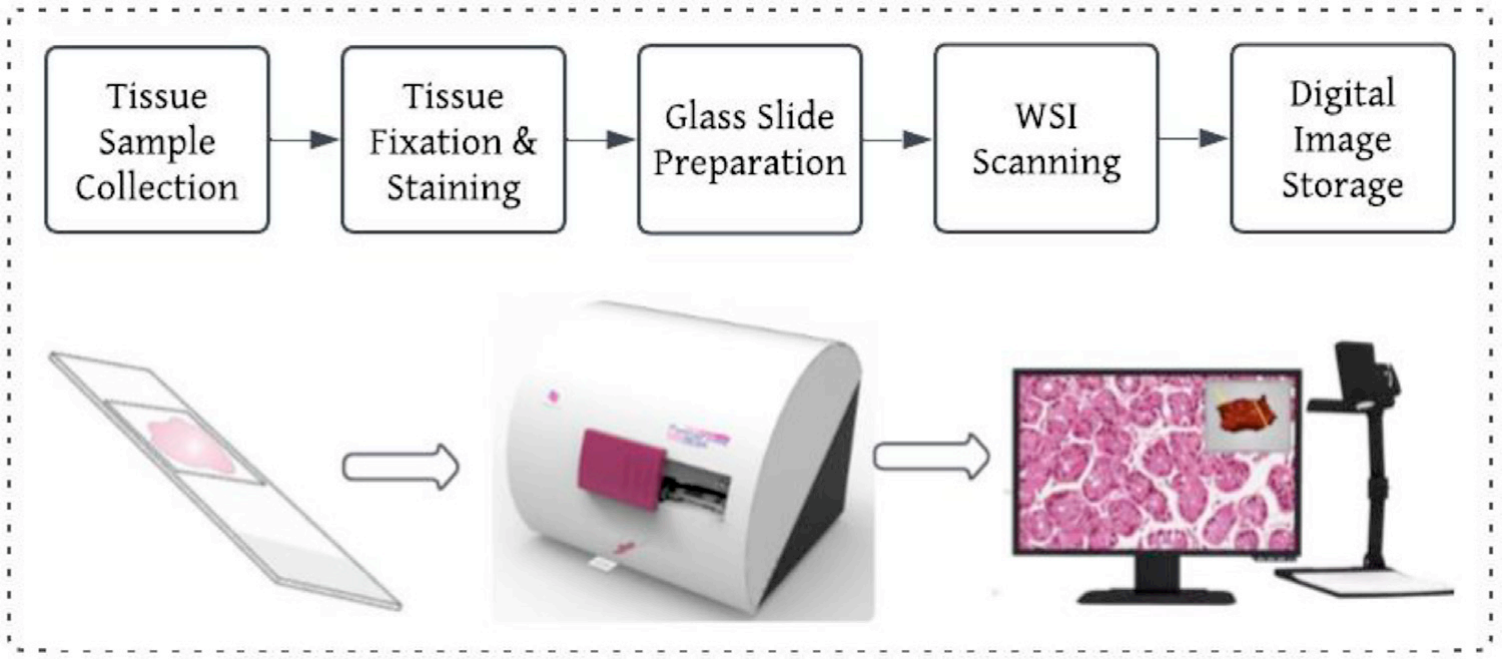
WSI scanning workflow and visual representation.

The HER2-IHC-40x dataset is derived from WSI of HER2 IHC-stained breast cancer tissue sections scanned at 40 × magnification with a high-resolution whole-slide scanner to get the optimal possible visualization of HER2 expression patterns, as shown in Fig. 1. All the slides were scanned and stored in a standard format with high image fidelity for computational analysis.

The whole slide images were acquired using a high-resolution digital pathology scanner with optimized scanning parameters for the purposes of clearness and homogeneity. Scanning was performed at 40 × magnification such that tissue morphology and HER2-IHC protein expression can be viewed intimately. The digitized images were stored in the SVS format, which is widely utilized for whole-slide imaging due to its compatibility with many pathology analysis platforms, the detail scanning specification shown in Table 7.

**Table 7:** A structured summary of the scanning equipment and image properties is provided in the table below.

Parameter	Details
Scanner model	3DHistech Pannoramic DESK
Magnification	40x
File format	.svs
Image Dimensions	Variable (Tissue-dependent)
Color mode	RGB 24-bit

All of the WSIs scanned vary in image size depending on tissue section size. Resolution was optimized to include a high pixel population per micrometer so that even microscopic cellular arrangements were still easily recognizable. The images were stored in RGB 24-bit color format, preserving natural staining characteristics without the imposition of compression artifacts.

### 4.2 Scanning equipment and image specifications

All WSIs were scanned using the 3DHistech Pannoramic DESK scanner at 40× magnification to capture high-resolution tissue details essential for HER2 analysis. The slides were saved in .svs format with RGB 24-bit color to ensure compatibility and preserve staining quality. Table 7 summarizes the key specifications of the scanning setup used in this study.

### 4.3 Digitization and quality control

In order to offer consistent quality in all WSIs, a number of quality control measures were performed at the time of scanning. Automatic and manual focus correction was performed to minimize blurring caused by variations in tissue thickness. Color uniformity was also ensured across all images to prevent variation because of batch-to-batch staining variability. Scanning artifact-containing slides such as air bubbles, dust, or blurring were manually inspected and excluded from the dataset to maintain high image fidelity.

The final dataset was structured systematically, where each WSI was labeled according to its HER2 class and each patch according to HER2-score (0, 1+, 2+, 3+). The scanned slides were stored under a well-structured directory hierarchy so that convenient access was provided for further processing and machine learning tasks.

### 4.4 Region of interest (ROI) annotation and selection

**Fig. 2:**
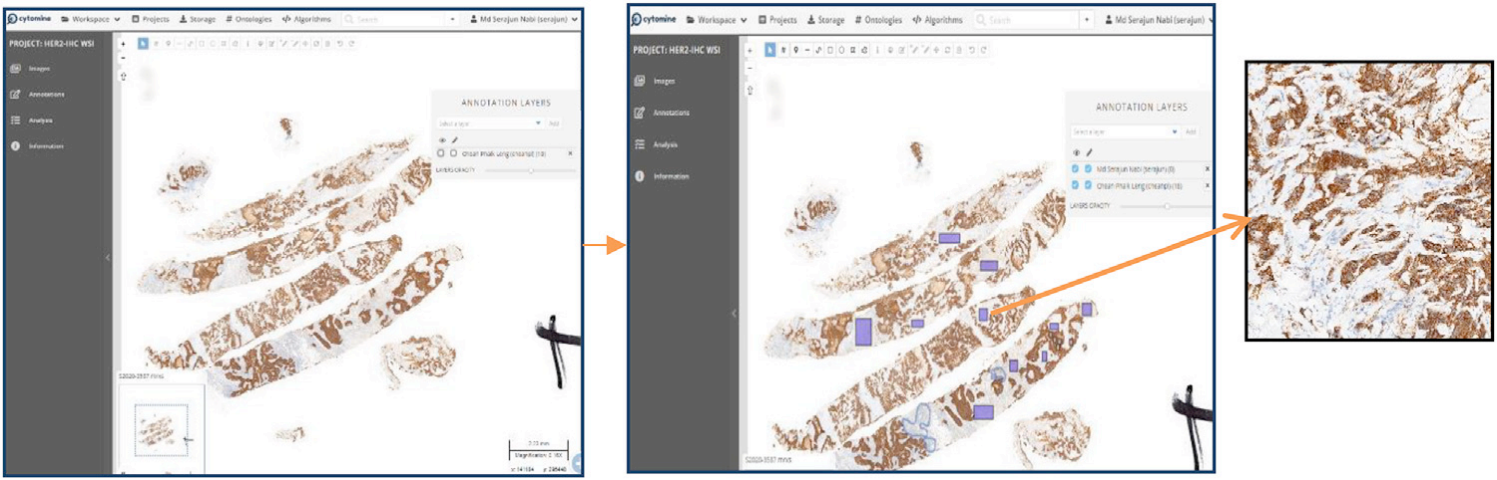
Cytomine Platform for Annotating Regions of Interest (ROI) in WSI.

After digitization of the Whole Slide Images (WSI), manual annotation and Region of Interest (ROI) selection were subsequently done to prioritize diagnostically relevant regions for HER2 classification. This was done by expert pathologists with the help of the Cytomine platform, a sample view in Fig. 2, which is a digital pathology software specifically created to ease collaborative annotation of WSIs.

### 4.5 Pathologist-guided annotation process

**Fig. 3:**
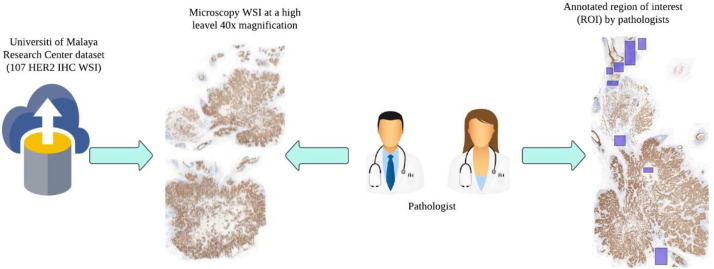
Pathologist-guided annotation of tumor regions in WSI using cytomine.

Each WSI contains a single large histopathological tissue section with varying scores of HER2 expression. To ensure that only tumor-relevant regions were included in the dataset, expert pathologists manually reviewed and annotated the slides using Cytomine, Fig. 3. Annotation was performed by outlining tumor-rich regions, ensuring that non-informative areas such as background, artifacts, and stroma were excluded.

Annotations were completed in multiple layers such that pathologists were able to distinguish between different HER2 expression regions within the same slide. This enabled the dataset to capture actual HER2 scoring groups (0, 1+, 2+, 3+) without mis-categorisation by irrelevant tissue regions.

### 4.6 ROI extraction and processing

Following annotation, the ROI patches chosen were exported from Cytomine for further structuring of the dataset. Each of the extracted ROIs was carefully reviewed to ensure that it met the following criteria:

• The region had a clear tumor presence with detectable HER2 staining.
• The ROI was free of any scanning artifacts (i.e., blurring, dust, or tissue folds).
• The extracted ROI size was normalized across different WSIs, making it consistent for use in machine learning.

The ROIs were then separated into single directories based on HER2 class labels, following the organized dataset hierarchy. The ROIs served as the basis for additional patch extraction, in which smaller high-resolution image patches were generated for model training and evaluation.

### 4.7 Patch extraction process

**Fig. 4:**
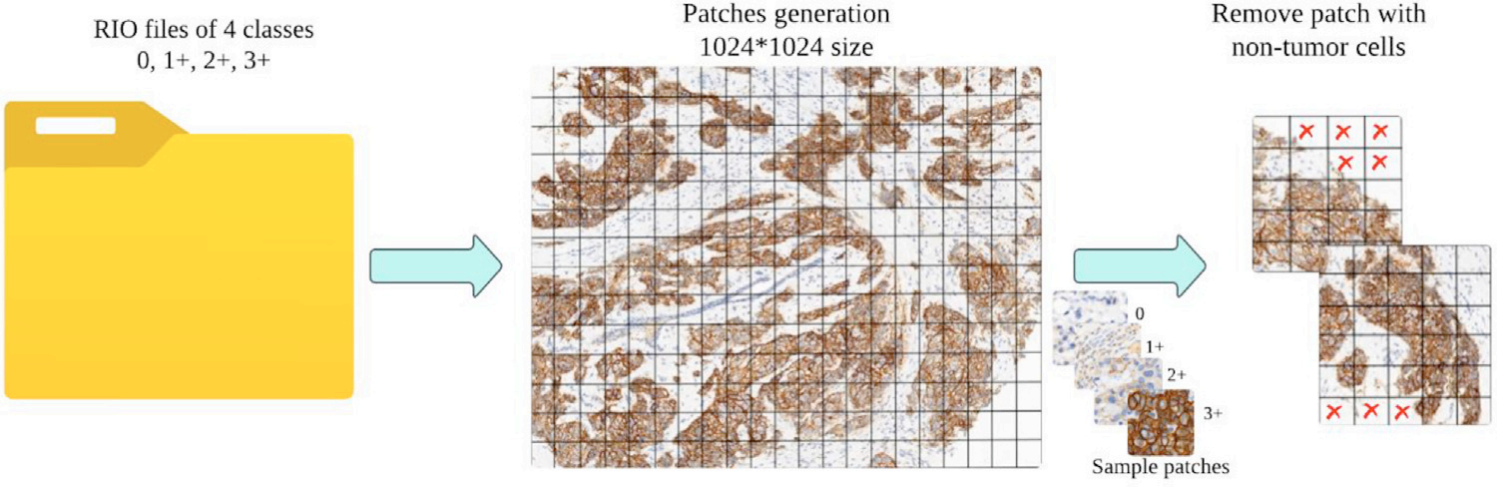
Patch extraction from annotated ROIs using adaptive methods.

Unlike traditional methods where patches are obtained through a static grid-based approach, this dataset required a more dynamic extraction process due to the different sizes of ROIs, Fig. 4. The process involved the following steps:

• ROI Input: All the labeled ROI images were processed as separate sources for patch generation. Since the ROIs had different sizes, direct grid-based segmentation was not applicable to all images.
• Adaptive Patch Extraction: Instead of a rigid non-overlapping grid, sliding window approaches were employed to extract multiple patches attempting to optimize the tissue content in each patch. This was performed to prevent patches from having informative tumor regions with HER2 staining.
• Patch Size Standardization: All patches retrieved were then resized to 1024 × 1024 pixels to maintain uniformity within the dataset.
• Patch Labeling: Each patch was assigned HER2 score of 0, 1+, 2+, or 3+ depending on the pathologist's original annotation of the source ROI.

### 4.8 Irrelevant patch removal and filtering

To ensure that only diagnostically significant patches were retained, a Hue-Saturation-Value (HSV) color histogram filtering method was employed [5]. This method filtered out non-tumor regions, staining artifacts, and excess background that would otherwise taint the classification model.

**Fig. 5:**
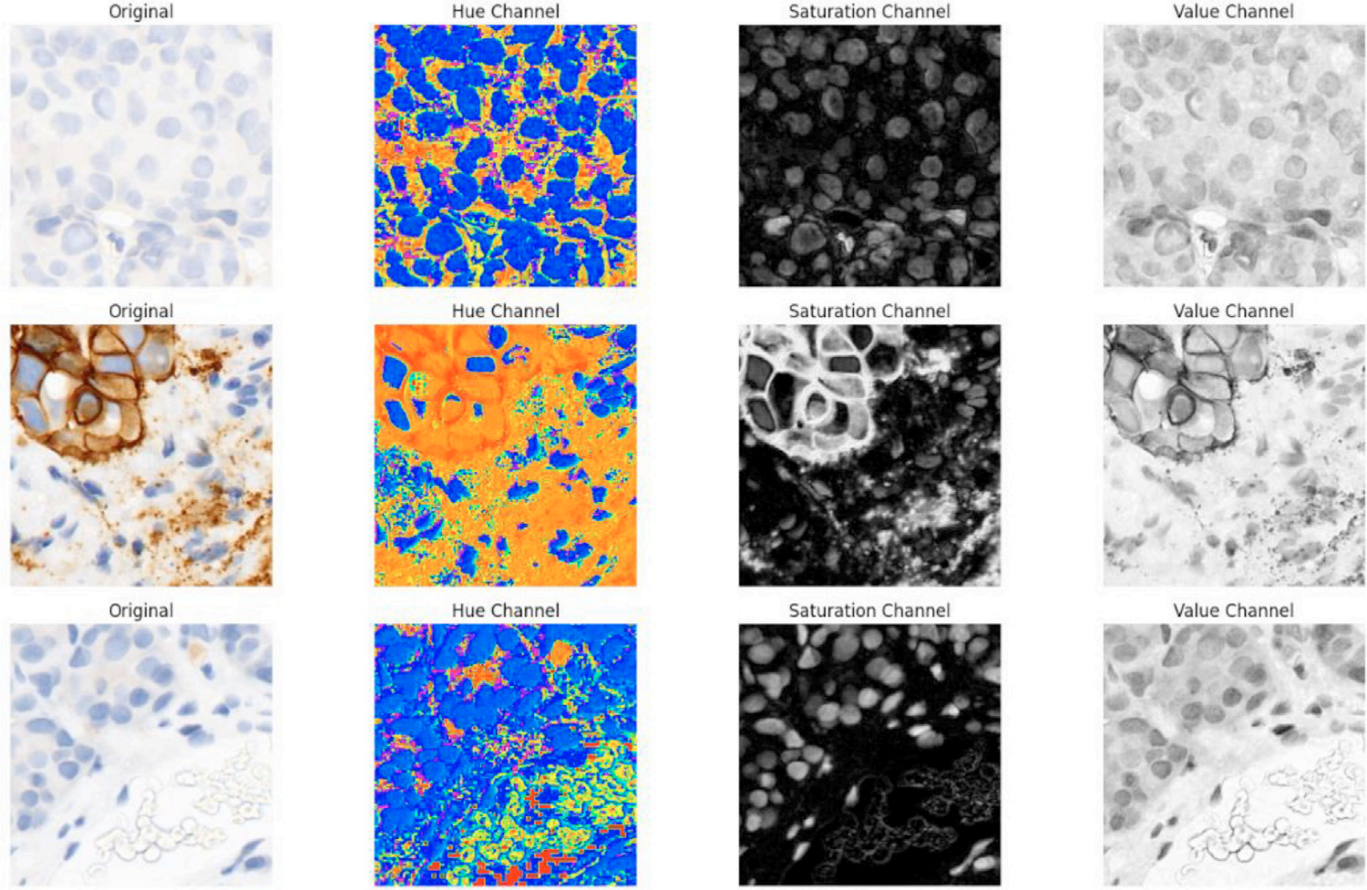
Color histogram analysis for excluding non-representative patches based on HSV channels.

HSV color model was employed because it represents colors in a way compatible with human perception, thus proving very effective in histopathology image processing [6]. Unlike the RGB model, which combines primary colors, HSV separates color description into three distinct components: Hue (H), the color type; Saturation (S), color intensity; and Value (V), brightness. By isolating the hue component, it became possible to differentiate informative HER2 staining from non-informative regions, and thus filtering could be performed more efficiently, a sample visualized output shown in Fig. 5.

To implement this method, reference histograms were first established by selecting 20 example images for each HER2 class (0, 1+, 2+, 3+). The patch for each was then converted from the RGB to the HSV color model, where its hue component was manipulated. A histogram was computed and normalized for each patch to have a common basis for comparison. The histograms calculated were then matched with the reference histograms using a measure of correlation. Patches that correlated poorly with their corresponding HER2 class were marked as non-representative and subsequently removed from the dataset.

**Fig. 6:**
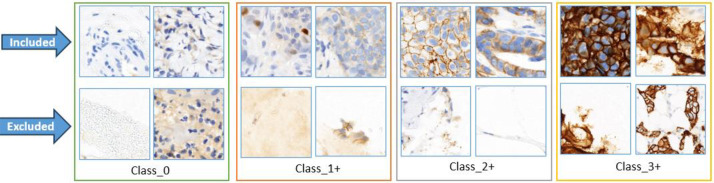
Examples of included and excluded patches based on color histogram filtering.

Patch hue histograms and class-specific reference histograms were compared using the Pearson correlation coefficient, which was applied using OpenCV's cv2.compareHist function and the HISTCMP_CORREL flag. Patches deemed to be poorly correlated were eliminated if their correlation score was less than 0.7. It was discovered that this empirically chosen cutoff was successful in keeping diagnostically significant tissue areas while eliminating non-representative patches like background, weak staining, or staining artefacts. Patches with high background or low contrast were eliminated, while those with strong HER2 staining patterns were included, as shown in Fig. 6. The quality and consistency of the dataset were greatly enhanced by this histogram-based filtering step, which created a solid foundation for deep learning-based HER2 IHC classification.

**Fig. 7:**
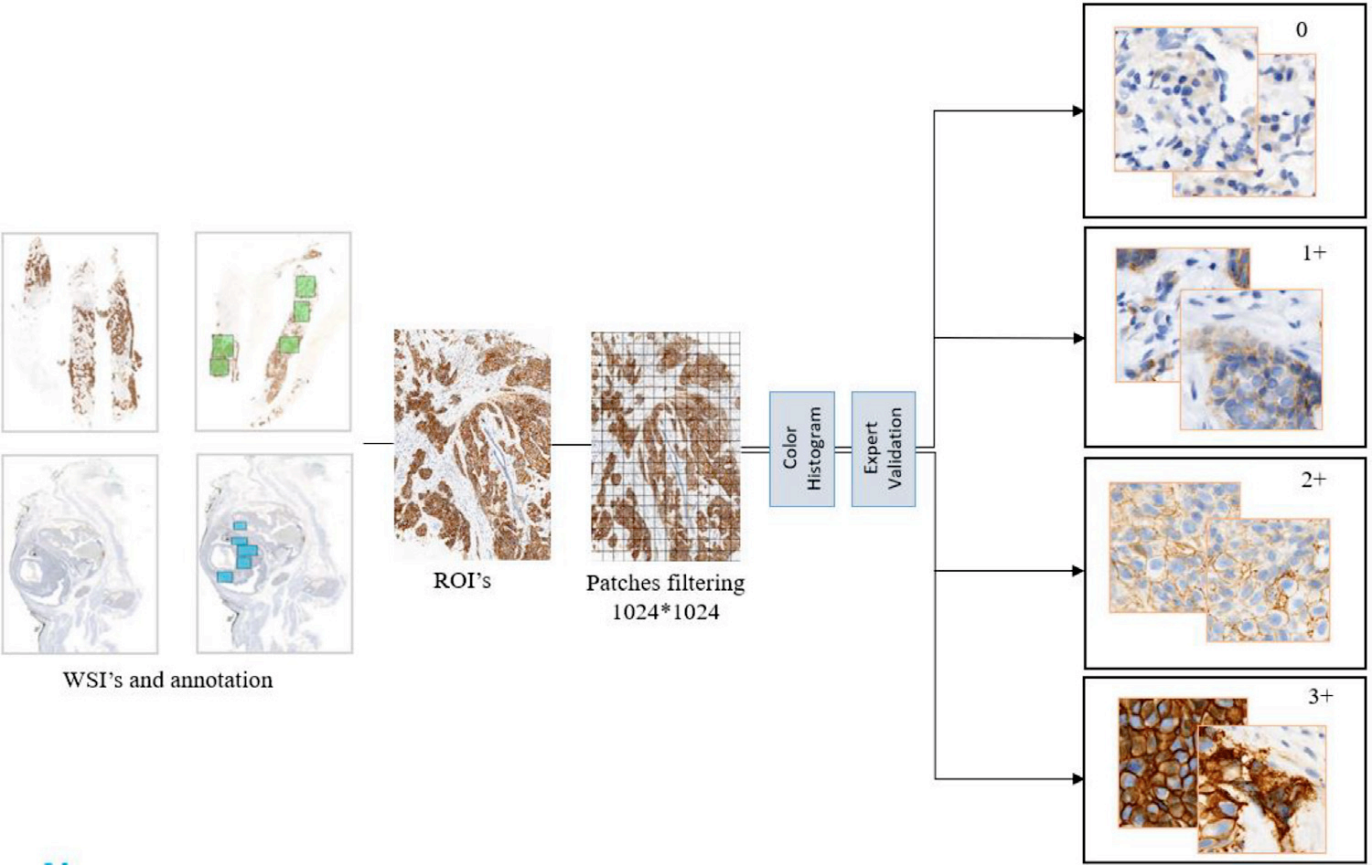
Overview of the data preprocessing pipeline from WSI to patch-Level HER2 scoring.

Through this color histogram-based filtering, the dataset quality was significantly improved such that all retained patches contained meaningful HER2 expression data. This rigorous approach enhanced dataset credibility for deep learning applications, providing a firm foundation for computational pathology studies. A final overview of HER-IHC-40x dataset preprocess pipeline shown in Fig. 7.

### 4.9 Software, tools, and computational environment

Dataset preparation involved processing of whole slide images, ROI annotation, patch extraction, and elimination of irrelevant patches, with a combination of Cytomine, OpenSlide, Python libraries, and Jupyter Notebook to run.

The Cytomine platform was used to annotate WSI, with pathologists manually selecting tumor-dense Regions of Interest (ROIs). Annotated ROIs were exported for processing. OpenSlide, an open-source library for WSI handling, was used to process and extract targeted ROIs from SVS files in an efficient manner.

Patch extraction from the ROIs was performed using OpenCV in Python, where 1024 × 1024-pixel patches were extracted. Other libraries such as NumPy and SciPy facilitated efficient image array handling and mathematical operations. Pandas were used for the structuring of the dataset so that patches were stored properly in respective HER2 classes, the summary shown in Table 8.

**Table 8:** Summary of tools and libraries used in data processing.

Category	Tools/Libraries Used
WSI Handling	OpenSlide
Annotation	Cytomine
Image Processing	OpenCV, NumPy, SciPy
Patch Extraction	OpenCV, Python
Histogram Filtering	OpenCV, SciPy, Matplotlib
Execution	Jupyter Notebook

### 4.10 Computational resources and hardware specifications

The dataset preparation was conducted on a system with the following hardware specifications:

As shown in Table 9, the GPU acceleration was leveraged for image processing tasks, particularly in batch-wise patch extraction and histogram computation, significantly reducing processing time.

**Table 9:** Computational resources and hardware specifications for dataset preparation.

Component	Details
Processor	Intel Core i7
RAM	16 GB or more
GPU	NVIDIA GTX 1080
Storage	SSD 512GB
Operating System	Windows 10

### 4.11 Code availability and dataset accessibility

The code scripts used for preprocessing, e.g., WSI processing, ROI extraction, patch generation, and color histogram-based filtering scripts, are openly available in the GitHub repository: https://github.com/seraju77/HER2-IHC-40x-data-preprocessing.git.

HER2-IHC-40x and HER2-IHC-40x-WSI has been hosted and deposited on Zenodo to offer long-term persistence and reproducibility. The dataset is publicly available through the following link: https://zenodo.org/records/15179608.

## Limitations

The HER2-IHC-40x dataset is limited in size, with 107 WSIs, which may not capture the entire variety of HER2 staining patterns. Although the ROIs were annotated by experienced pathologists, there may still be some degree of heterogeneity in the interpretation of staining. Even after applying color histogram filtering, faint staining artifacts and background noise may remain. Additionally, since the dataset is from a single institutional source, potential biases due to staining protocols and scanning conditions would undermine generalizability.

## Ethics Statement

The authors have read and follow the ethical requirements for publication in Data in Brief and confirming that the current work does not involve human subjects, animal experiments, or any data collected from social media platforms.

## Credit Author Statement

**Md Serajun Nabi:** Conceptualization, Data curation, Formal analysis, Methodology, Resources, Software, Visualization, Writing - original draft, Writing - review & editing. **Mohammad Faizal Ahmad Fauzi, and Hezerul Bin Abdul Karim:** Conceptualization, Data curation, Formal analysis, Funding acquisition, Investigation, Methodology, Project administration, Supervision, & Writing - review & editing. **Phaik Leng Cheah, Seow Fan Chiew, and Lai Meng Looi:** Validation.

## Data Availability

ZenodoHER2-IHC-40x: A High-Resolution Histopathology Dataset for HER2 IHC Scoring in Breast Cancer (Original data).
